# Supplementary material for: Distributed neural representation of saliency controlled value and category during anticipation of rewards and punishments
Source: Nat Commun. 2017 Dec 4;8:1907. doi: 10.1038/s41467-017-02080-4 (PMC5714958; doi:10.1038/s41467-017-02080-4)
Supplement: Supplementary file 1 — Supplementary Information [file 41467_2017_2080_MOESM1_ESM.pdf]

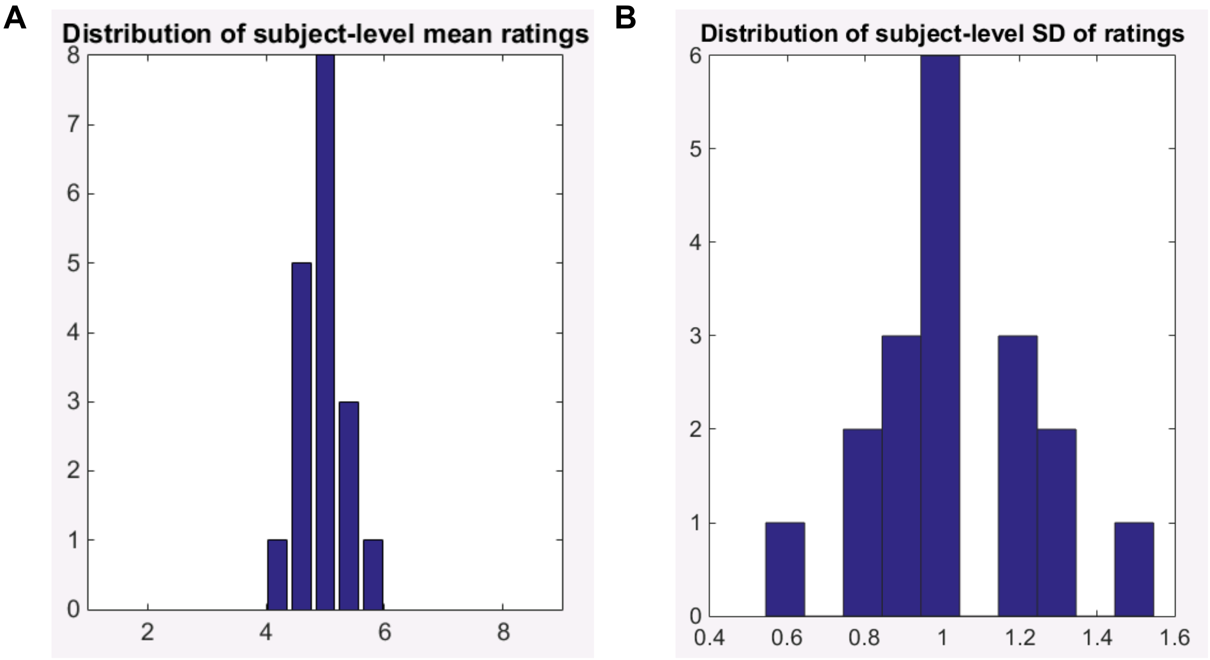

Supplementary Figure 1. (*Related to Figures 1D and 3)* **Individual-level distribution characteristics of pleasantness ratings**

A. Histogram of individual-level mean cue ratings across all categories.

B. Histogram of individual-level standard deviation of ratings across all categories.

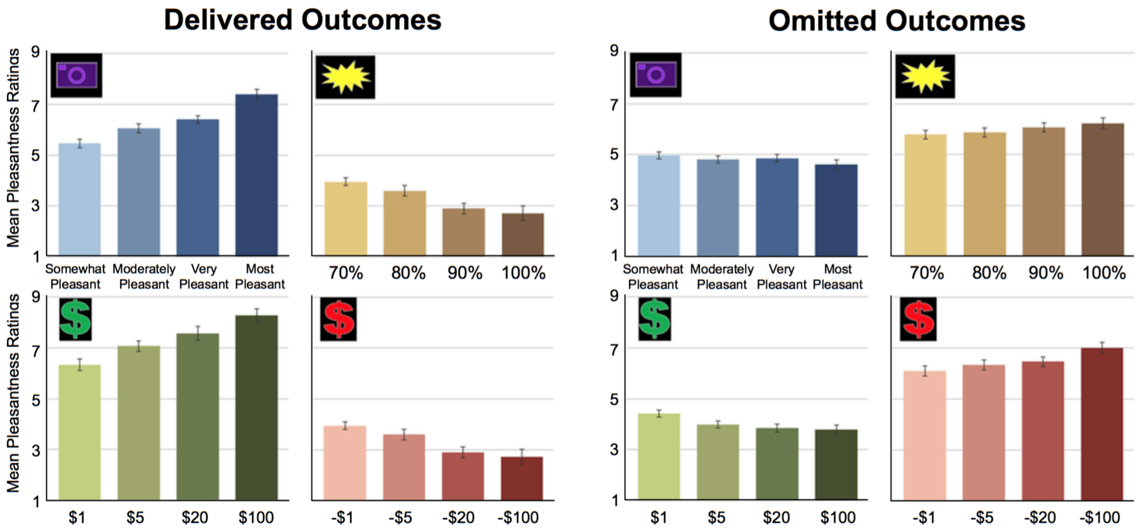

Supplementary Figure 2. (*Related to Figure 1D)* **Pleasantness ratings for the outcome period**

A. Pleasantness ratings of delivered outcomes of different levels and of different categories.

B. Pleasantness ratings of omitted outcomes of different levels and of different categories.

Mean ratings were from averaging across 18 participants, and error bars denote SEM.

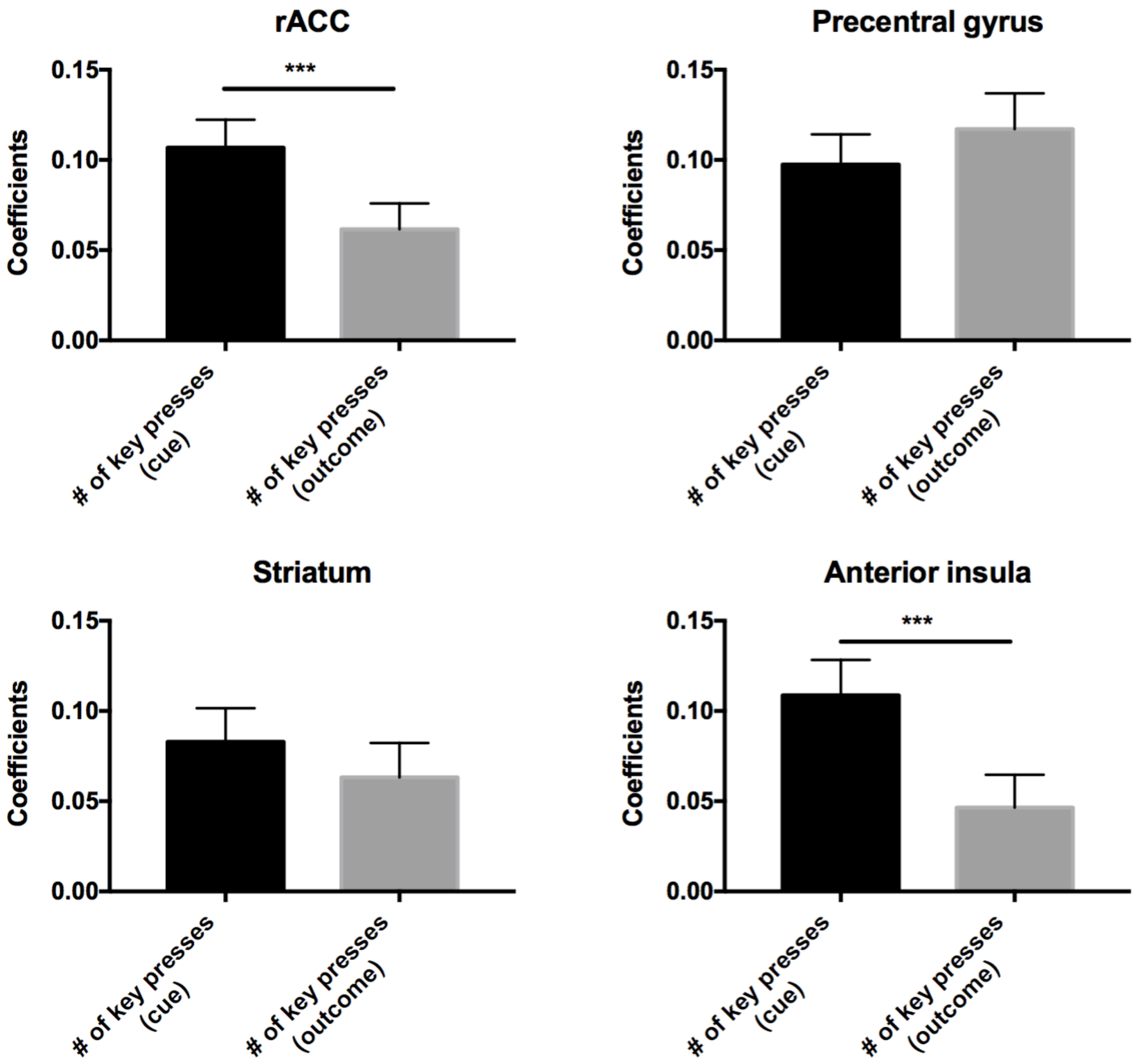

Supplementary Figure 3 (*Related to Figure 3)* **Hand movement analysis in saliency regions**

Bars indicate mean regression coefficients for the number of key press predictor in the ROI GLM analyses. Asterisks indicate significance of paired *t* tests (***, $p < 0.001$).

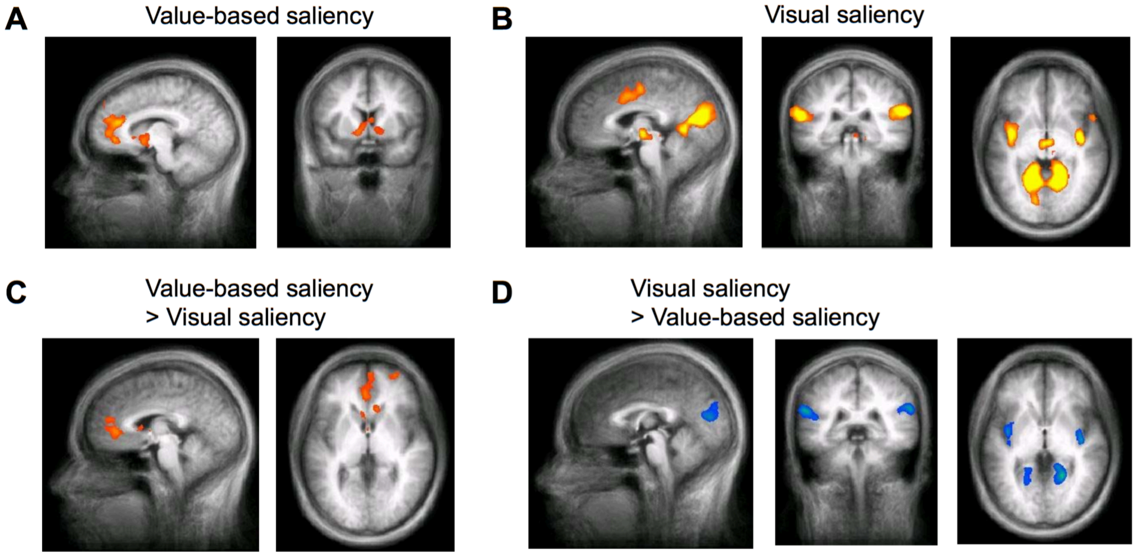

Supplementary Figure 4 (*Related to Figure 3)* **Brain regions responding to value-based saliency vs. visual saliency**

A. Value-based saliency (per-voxel $p < 0.005$, cluster-size thresholding with simulation of 1000 iterations, FWE $p < 0.05$, threshold = 92 voxels).

B. Visual saliency (per-voxel $p < 0.005$, cluster-size thresholding with simulation of 1000 iterations, FWE $p < 0.05$, threshold = 98 voxels).

C,D. Value-based saliency vs. visual saliency (per-voxel $p < 0.005$ uncorrected, cluster size > 20 voxels). Yellow indicates value-based saliency > visual saliency, while blue indicates the opposite direction.

A Monetary gains > others

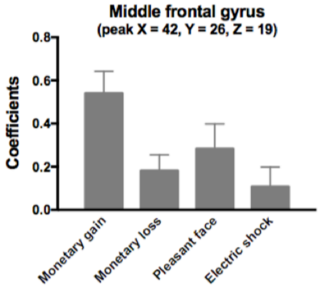

B Pleasant faces > others

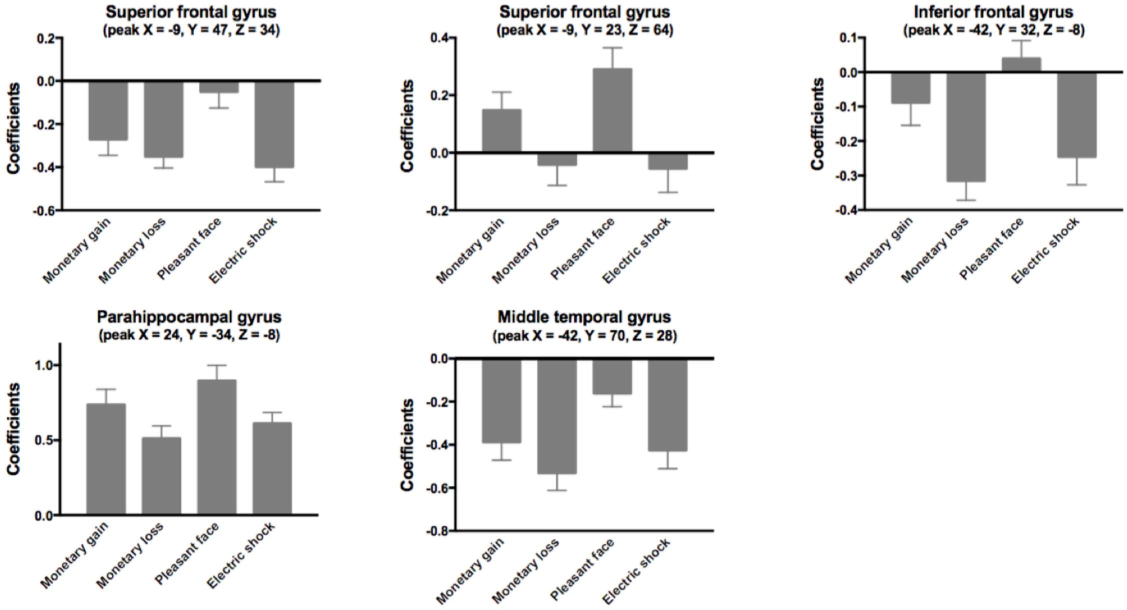

C Electric shocks > others

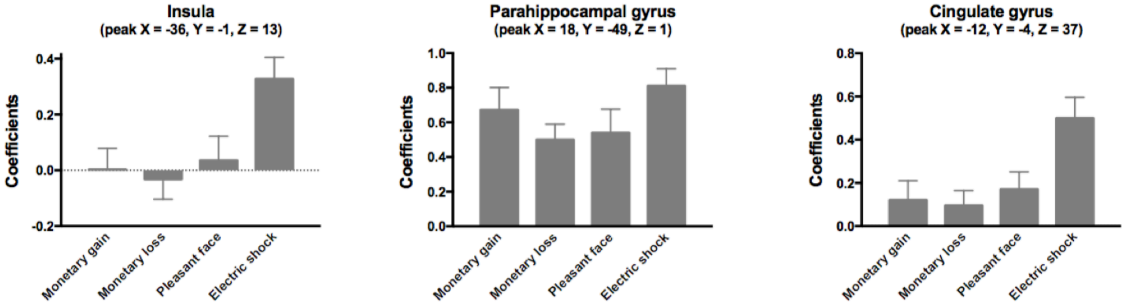

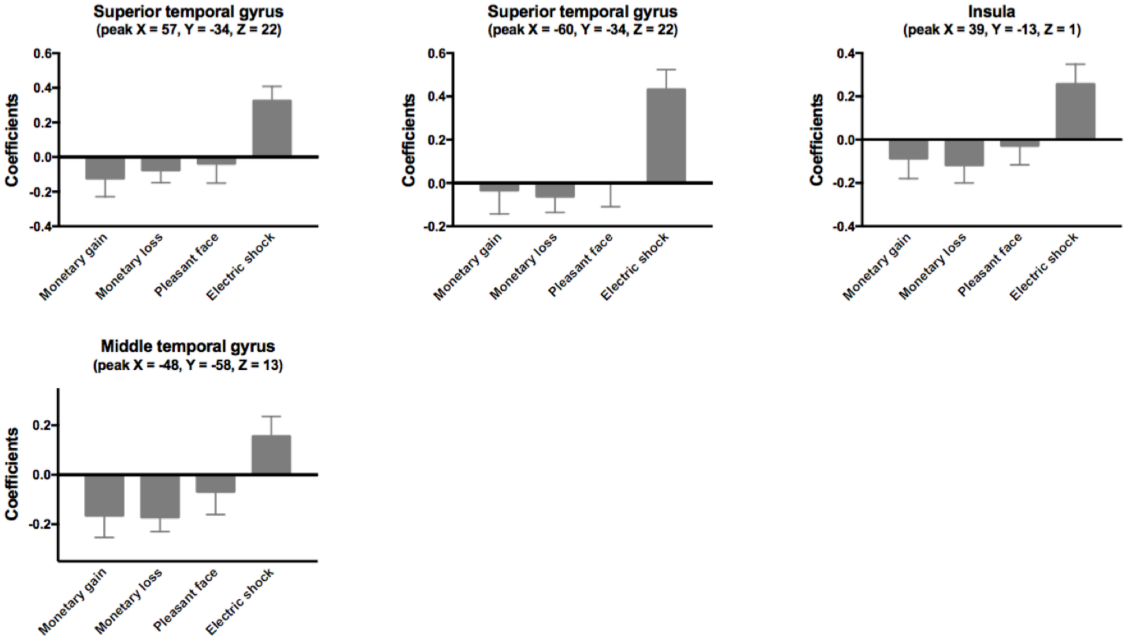

Supplementary Figure 5 (*Related to Supplementary Table 3)* **Category specificity of univariate representations of category identities**

Only non-visual brain regions are plotted here. No region outside of the occipital cortex showed increased activation for monetary losses compared with the other three categories. See Table 2 of the main text for additional information on these regions.

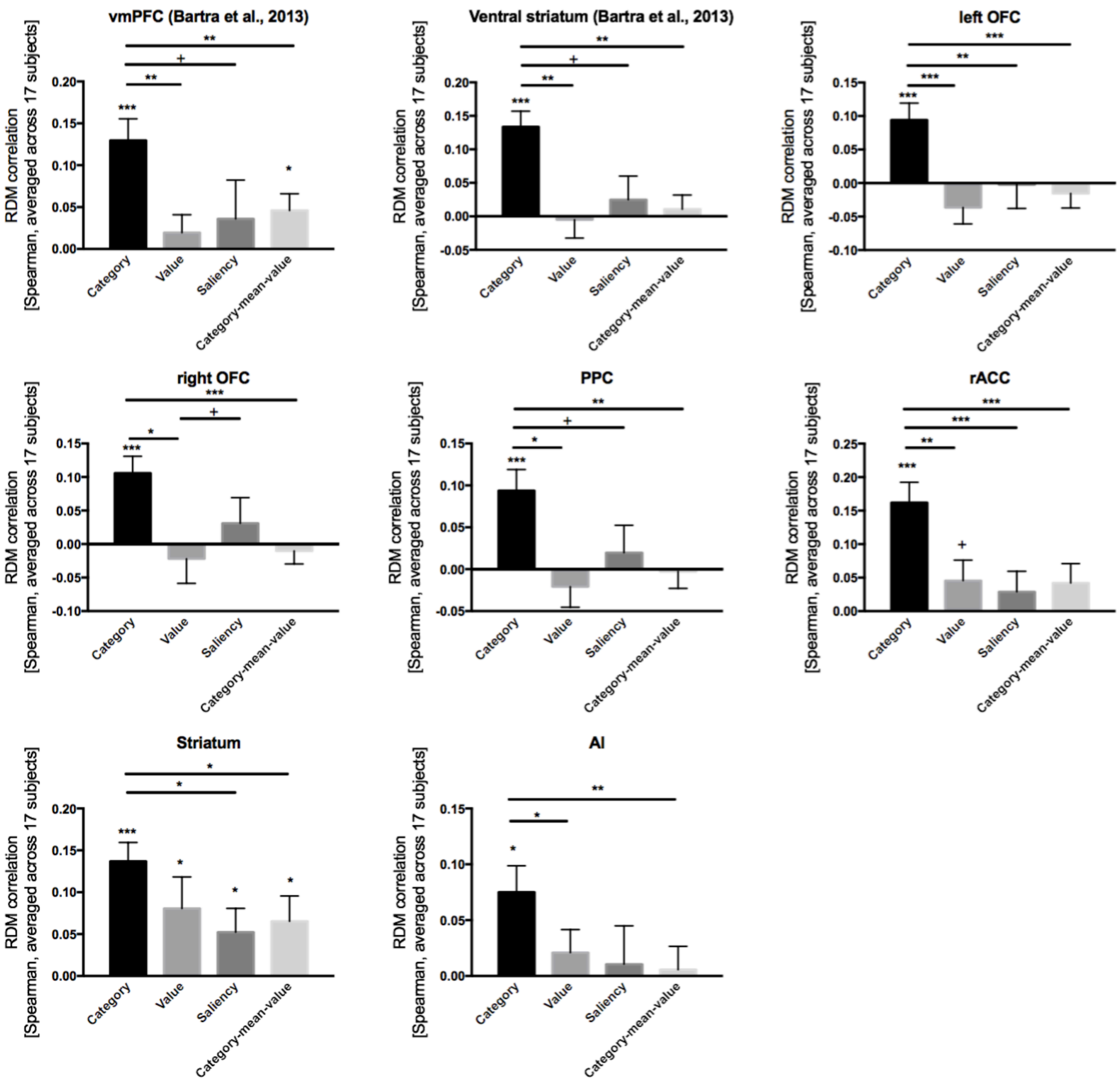

Supplementary Figure 6 *(Related to Figures 2-4)* **Region-of-interest (ROI) RSA for value and saliency regions in the univariate analysis**

Results from RSA on ROIs identified in univariate GLM analysis (see Figure 2). Mean Spearmen correlation coefficients between the candidate models and the neural RDM averaged across 17 participants are represented by bars, and error bars stand for standard error of the mean (S.E.M.). Significance of individual models are labeled on top of the bars. Horizontal bars at the top of the plots indicate significant (or marginally significant) pairwise comparisons between two models. +, p <0.1; *, p < 0.05; **, p < 0.01; ***, p < 0.001. All p values were false discovery rate (FDR) corrected.

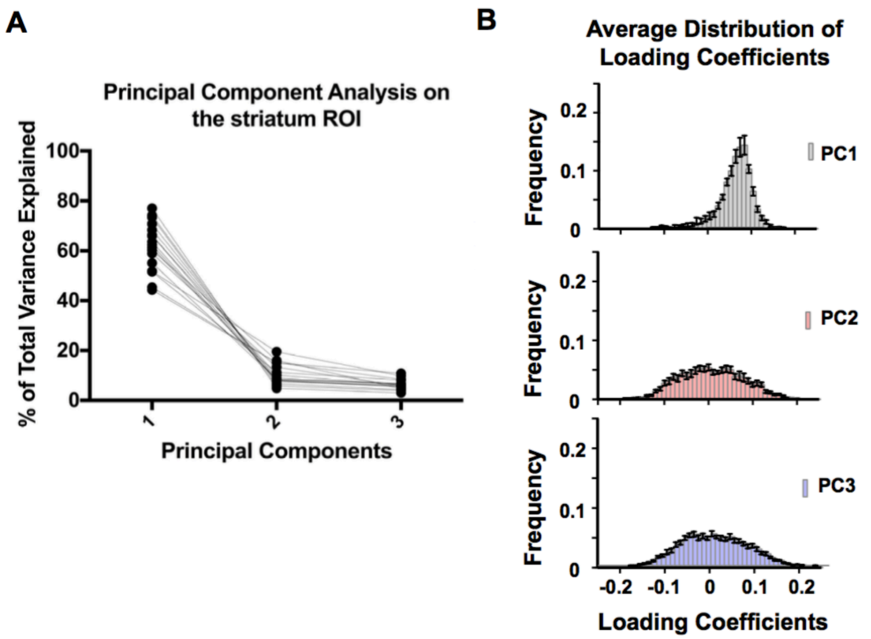

C

Principal component regression

	Value	Saliency	Category
PC1	√ (*p* = 0.03)	×	×
PC2	×	×	×
PC3	√ (*p* = 0.09)	×	√ (monetary gain *p* = 0.04; pleasant face *p* = 0.09)

Supplementary Figure 7 *(Related to Figure* 7*)* **Principal component analysis of ensemble patterns in ventral striatum**

A. Percentages of total variance explained by the first 5 PCs in each participant. Each dot is a data point from a participant, and data from the same participant are connected by dashed lines.

B. Average histograms of loading coefficients for the first 3PCs. The frequency of loading coefficients falling under a particular bin was calculated separately for each participant. The mean and the SEM of frequencies in this bin were then obtained. This was repeated for all bins, spanning the entire range of loading coefficients. The mean frequencies in each bin are plotted here in the form of a histogram with error bars representing SEMs.

C. Summary of results of mixed-effect linear regression of the first 3 PCs on value, saliency, and category.

Supplementary Table 1. Brain regions that showed value-based or visual saliency responses during the cue period.

Contrast	Region	Side	mean *t* statistic	Peak Talairach coordinates			Cluster size (number of voxels)
				x	y	z	
Value-based saliency	rACC	L/R	3.94	-3	44	7	336
	Precentral gyrus	L	3.98	34	36	2	203
	Striatum	L/R	3.59	-9	5	-5	124
	Cuneus	R	3.61	15	-67	4	103
	Middle occipital cortex	R	3.62	39	-67	-2	105
Visual saliency	Cuneus	L/R	4.72	-9	-73	7	1639
	Superior temporal gyrus	L	3.90	-57	-37	22	348
	Superior temporal gyrus	R	3.90	57	-31	22	306
	Posterior insula	R	3.69	36	-7	-2	239
	Posterior insula	L	3.60	39	-7	-2	223
	Midbrain/thalamus	L/R	3.61	-3	-13	-5	135
	Middle cingulate cortex	L/R	3.31	3	-10	46	189
Value-based saliency > Visual saliency	rACC	L/R	3.65	-3	38	4	174
	Striatum	L/R	3.69	0	8	10	97
Visual saliency > Value-based saliency	Superior temporal gyrus	L	4.17	-57	-34	22	124
	Poterior insula	L	3.78	-39	-4	-2	54
	Poterior insula	R	4.00	39	-13	1	41
	Superior temporal gyrus	R	3.85	57	-34	22	95
	Cuneus	L/R	3.78	0	-76	13	134

For the first two contrasts, simulation-based cluster size thresholding was applied on the statistical (*t*) map (per-voxel $p < 0.005$, cluster-size thresholding with simulation of 1000 iterations, FWE $p < 0.05$, threshold = 92 voxels for value-based saliency and 98 voxels for visual saliency). All clusters evolving from such thresholding were reported. For the last two contrasts, a more liberal threshold was used (per voxel $p < 0.005$ uncorrected, cluster size > 20 voxels). Because of the lower threshold, a larger number of clusters evolved from the contrast (12 for value-based saliency > visual saliency and 7 for visual saliency > value-based saliency). For brevity, only clusters related to the ones found in the first two contrasts were included in this table.

Supplementary Table 2. Brain regions that showed univariate responses for category valence, for category modality, and for their interactions.

Effect	Region	Side	mean *F* statistic	Peak Talairach coordinates			Cluster size (number of voxels)
				x	y	z	
Valence main effect (Positive vs. Negative)	Inferior frontal gyrus	L	16.95	-47	34	2	84
	Lingual gyrus	L	14.19	-19	-64	-3	57
	Parahippocampal gyrus	R	14.10	30	-44	-3	60
	Middle frontal gyrus	L	12.86	-33	12	53	67
Modality main effect (Primary vs. Secondary)	Inferior occipital gyrus	R	20.37	40	-79	-1	1166
	Middle occipital gyrus	L	17.06	-29	-80	-8	1000
	Inferior parietal lobule	L	15.30	41	-51	55	65
	Precuneus	R	15.94	18	-77	36	95
	Superior temporal gyrus	L	13.86	-55	-33	19	338
	Lentiform nucleus	L	13.89	-22	-8	-1	116
	Superior temporal gyrus	R	14.85	53	-39	20	50
	Caudate	R	14.59	16	15	7	75
Valence × Modality interaction	Superior temporal gyrus	L	21.72	-58	-39	21	197
	Lingual gyrus	L	20.64	-22	-58	4	975
	Insula	L	14.44	-40	-4	14	128
	Middle temporal gyrus	L	13.26	-56	-61	12	52
	Cingulate gyrus	L	12.95	-7	5	34	104

Supplementary Table 3. Brain regions that showed univariate responses for the identity of specific categories during the cue period.

Contrast	Region	Side	mean *t* statistic	Peak Talairach coordinates			Cluster size (number of voxels)
				x	y	z	
Monetary gain > (Monetary loss + Pleasant face + Electric shock)	Tuber	L	3.89	-43	-72	-30	163
	Tuber	R	3.72	31	-60	-30	77
	Middle frontal gyrus	R	3.89	46	21	21	39
	Cuneus	R	3.71	19	-75	9	106
	Inferior occipital gyrus	R	3.71	40	-77	-1	70
Monetary loss > (Monetary gain + Pleasant face + Electric shock)	Lingual gyrus	L	4.33	-7	-81	-3	48
	Cuneus	L	3.98	11	-95	-4	89
	Inferior occipital gyrus	L	3.85	-40	-70	-3	39
	Inferior occipital gyrus	R	3.76	34	-79	-1	108
Pleasant face > (Monetary gain + Monetary loss + Electric shock)	Superior frontal gyrus	L	3.45	-12	46	31	46
	Superior frontal gyrus	L	3.59	-12	22	57	36
	Inferior frontal gyrus	L	3.43	-46	28	123	123
	Parahippocampal gyrus	R	3.30	24	-34	34	34
	Middle temporal gyrus	L	3.16	-42	-69	56	56
Electric shock > (Monetary gain + Monetary loss + Pleasant face)	Insula	L	3.76	-38	-4	16	159
	Parahippocampal gyrus	R	3.76	18	-50	4	64
	Cingulate gyrus	L	3.68	-13	-4	34	83
	Lingual gyrus	L	3.68	-15	-48	2	141
	Superior temporal gyrus	R	3.64	56	-39	20	113
	Superior temporal gyrus	L	3.53	-58	-36	22	160
	Cuneus	L	3.31	-7	-80	12	67
	Insula	R	3.37	40	-17	6	57
	Middle temporal gyrus	L	3.15	-48	-59	14	34

Supplementary Table 4 Brain regions with statistically significant effects of the additional control models for category encoding in the whole-brain searchlight RSA analysis

Candidate model	Region	Side	mean *z* statistic	Peak Talairach coordinates			Cluster size (number of voxels)
				x	y	z	
Category-mean-value	Lingual gyrus	L/R	3.24	12	-78	7	1097
	Paracentral lobule	L/R	3.07	0	-27	48	41
Primary-secondary	Inferior parietal lobule	R	3.10	42	-36	29	236
	Lingual gyrus	R	3.04	18	-79	-1	169
	Parahippocampal gyrus	R	3.09	23	-36	-7	101
	Middle occipital gyrus	L	3.06	-40	-78	5	301
	Middle frontal gyrus	R	3.03	41	31	28	79
	Medial frontal gyrus	L/R	3.06	12	43	28	296
	Superior temporal gyrus	L	3.02	-55	-36	22	99
Positve-negative	Precuneus	L/R	3.21	0	-62	5	1227
	Cingulate gyrus	L/R	3.11	-3	-21	36	145
	Posterior insula	R	3.08	37	-11	6	62
	Middle frontal gyrus	R	2.99	40	22	34	80
	Cingulate gyrus	R	3.08	3	20	35	60
	Superior temporal gyrus	L	3.10	-58	-31	17	137
	Superior temporal gyrus	R	3.02	53	-36	18	118
Money vs. face vs. shock	Middle frontal gyrus	L	3.30	39	41	13	131
	Superior temporal gyrus	R	3.34	54	37	-22	223
	Middle temporal gyrus	R	3.28	39	-67	22	146
	Precentral gyrus	R	3.11	48	-1	13	304
	Parahippocampal gyrus	R	3.00	27	-49	08	81
Monetary gains vs. losses vs. primary	Precuneus/occipital lobe	L/R	3.16	3	-65	2	572

Supplementary Note 1: Calculation of saliency

Our main analysis assumed the same predefined neutral rating (5) for all participants. A potential concern is that different participants may use different neutral points in their value estimations. Here we elaborate on the rationale for this choice and contrast it with alternative options, such as mean centering and normalization.

First, as explained in the main text, when we introduced the rating scale to the participants during the pre-scan instructions, we did provide a standard interpretation of the ratings by emphasizing that 5 was neutral, and anything above/below 5 would be pleasant/unpleasant (these interpretations also appeared on the screen next to the scale in every trial). Admittedly, there is no objective way of verifying that all subjects used the scale in the way we asked them to, but it seems appropriately conservative to assume so without further information of a systematic bias. Second, in both mean centering and normalization, using the mean rating as the neutral point also implicitly assumes that overall the positive and negative outcomes in our paradigm cancelled each other out in terms of deviations from neutrality, which may not necessarily be true. To illustrate this, let us imagine a participant who uses the rating scale with 5 as her subjective neutral point, and gives monetary gains, monetary losses, and pleasant faces average ratings of 7, 3, and 7, respectively. Meanwhile, this participant is particularly sensitive to pain, so the electric shocks are more negative (and more salient) to her. As a result, her average rating for electric shocks is 1.5 (still with 5 being neutral). Under such circumstance, the mean rating across all categories for this subject will be below 5, but using this mean as the neutral point to calculate saliency would introduce much bias – the saliency for the two

negative categories would be lower, while the two positive categories would have inflated saliency.

With such caveat in mind, we can also examine the distribution of subject-level mean ratings in our behavioral data, to search for participants (if any) who only reported very high or very low ratings (Supplementary Figure 1A). It can be seen that the subject-level mean ratings were indeed distributed within a relatively narrow range around 5, and the 'mean of the mean' was 4.93. This indicates that our dataset did not have extreme cases where only one end of the scale was used (e.g. a 'pessimistic' subject using 5 as the highest response).

In addition to mean centering, normalization also involves rescaling centered data by the standard deviation, which essentially means adjusting the saliency by the range of ratings of individual participants in this context. The distribution of subject-level standard deviations of ratings is presented in Supplementary Figure 1B, where a moderate degree of individual differences can be observed. It is important to recognize the two potential sources of such differences: individual differences in the usage of the scale and/or individual differences in stimuli/outcome evaluation. For example, two subjects, A and B, had ratings ranging from 1 to 9 and 3 to 7, respectively. While it is possible that the two of them had the same experience in the task and only differed in how they used the rating scale, it is also possible that subject A did have a more intense affective experience (thereby having a greater dynamic range for ratings) than subject B. In the latter case, assuming that a rating of 9 for A indicates the same level of pleasantness and salience as a rating of 7 for B would be detrimental for examining the neural representation of these quantities across subjects.

To summarize, calculating saliency by mean centering or normalization (z-scoring) entails making a set of strong assumptions of how ratings map onto subjective value/saliency, which are hard to verify. More broadly speaking, these issues are general limitations of working with ratings. We believe that our approach is a more conservative choice given how the experiment was designed and administered, as well as the participants' behavior. It is important that readers evaluate our results with these caveats in mind.

Supplementary Note 2: Excluding the potential confound of hand movement for the univariate saliency signal

Since participants needed to move the cursor from the central starting position on the scale to the desired number, the number of button presses increased as the rating deviated more from the neutral rating 5. As a result, saliency estimates based on participants' trial-by-trial ratings were strongly correlated with hand movement and/or the time it took to register the response. Indeed, among the saliency regions was a part of left precentral gyrus (see Table 1), whose location corresponded well with the right hand area of the primary motor cortex. It is critical that we are able to exclude the possibility that the saliency signals we found are merely an artifact of hand movements or reaction time.

If the saliency signals we observed were driven by these potential confounds, however, then we should expect to see similar signals both during the cue period and during the outcome period, as identical rating procedures were used in both. Although the cognitive processes could be different in the cue period and in the outcome period, the process of motor control should be the same. Moreover, the dynamic ranges of the ratings for cues

and of the ratings of outcomes were very similar (compare Figure 1 and Supplementary Figure 2), also facilitating a fair comparison between the two periods. To examine this possibility we constructed a GLM (see Supplementary Methods for details) which directly modeled the minimum required numbers of key presses in both periods on a trial-by-trial basis. We then fit this GLM to the voxel-wise mean BOLD time course of the saliency ROIs, and compared the regression coefficients for the number of key presses in the cue and outcome periods. If the activity observed in saliency areas is entirely due to movement, then there should be no difference between the two phases, as the neural process related to movement should be entirely the same. Conversely, if there was significant difference between the two coefficients, then it would be hard to ascribe the observed neural activity *purely* to hand movements, because it was unlikely that neural activity controlling hand movement would be modulated by task period in this context.

Paired t tests in each of the four saliency ROIs showed that in both rACC and anterior insula, the differences were highly significant (rACC p = 0.00086, anterior insula p = 0.00045; Supplementary Figure 3), while in striatum and in precentral gyrus there was no significant difference (precentral gyrus p = 0.34, striatum p = 0.23; Supplementary Figure 3).

The activities of rACC and insula were thus clearly inconsistent with a movement account, while activity in the precentral gyrus (acting as a positive control) was, in line with previous knowledge about this area. The difference also did not reach significance in striatum, probably because the striatum subserves both motor and cognitive functions. It must be pointed out, however, that even with this, it is not necessarily true that the striatum or the precentral gyrus do not signal saliency. Because of the correlation

between saliency and movement, it remains possible that these regions are responding to saliency in *both* cue and outcome periods. Finding a definitive answer to this question is important but falls outside of the scope of our study.

Supplementary Note 3: Separating the neural substrates of value-based saliency and visual saliency

Value-based saliency is defined by the deviation of an outcome's subjective desirability from the neutral reference point, so that both very positive and very negative outcomes (and cues predicting them) have high saliency. Meanwhile, saliency can also be driven by the sensory properties of a stimulus. In this case, saliency is defined by the prominence of a stimulus in the perceptual space. Our operationalization of saliency in this study likely encompasses both kinds of saliency, because cues that predicted more appetitive/aversive outcomes were also visually more conspicuous. The saliency signals we identified in the main GLM analysis (Table 1 and Figure 3) may therefore reflect value-based saliency, visual saliency, or both. Below we outline further analysis that aimed at distinguishing these two different sources of saliency.

To quantify the visual saliency of each of the 16 visual stimuli in our experiment, we used a prominent visual saliency model proposed in the context of computational modeling of human visual perception (66), to generate estimates of the saliency of the cue stimuli (see SI methods for details). With these visual saliency estimates, we then constructed a new general linear model (GLM) for controlling the potential confound of visual saliency. This GLM was generated by adding the visual saliency estimates as one

additional parametric modulator for the cue period (after convolution with a standard canonical hemodynamic response function) to the main GLM used for the search of value and (value-based) saliency signals in the whole brain. Therefore, for non-actualized trials, the cue was modeled by a binary regressor and *three* parametric regressors modulated by trial-by-trial (1) value, (2) value-based saliency, and (3) visual saliency estimates. This new GLM enabled us to examine neural activations related to either value-based saliency or visual saliency, while controlling for the other type of saliency.

To address the potential confound of visual saliency, we performed two complementary analyses with this new GLM - a whole-brain search for representations of the two saliency signals and a region-of-interest (ROI) analysis. First, we performed a whole-brain search for significant coefficients of the value-based saliency and visual saliency predictors. Using the same statistical threshold (per-voxel $p < 0.005$, cluster-size thresholding, FWE $p < 0.05$, threshold = 92 voxels) as before, for correlations with the value-based saliency predictor, we were still able to recover most value-based saliency regions we reported, including rACC, striatum, and precentral gyrus (Supplementary Figure 4A and Supplementary Table 1, and compare with Figure 3 and Table 1 in the main text). We also found bilateral anterior insula activations to the value-based saliency predictor, albeit in smaller clusters (left: 40 voxels; right: 86 voxels), which did not survive the cluster thresholding. Interestingly, the widespread activation in visual cortex observed in the original analysis (see Figure 3 in the main text) was much diminished. Conversely, examining the correlation with the visual saliency predictor (per-voxel $p <$ 0.005, cluster-size thresholding, FWE $p < 0.05$, threshold = 98 voxels) revealed clusters in the visual areas, as well as bilateral superior temporal gyrus, bilateral posterior insula,

midbrain/thalamus, and middle cingulate cortex (Supplementary Figure 4B and Supplementary Table 1). A direct contrast between value-based saliency and visual saliency also confirmed the findings above, although at a slightly more liberal statistical threshold (per-voxel $p < 0.005$ uncorrected, cluster size > 20 voxels). Thus, after controlling for visual saliency, activity in the value-based saliency regions we previously reported (rACC, striatum, and anterior insula) was significantly correlated with value-based saliency, while activity in a separate set of brain regions was correlated with visual saliency.

Second, we examined the main value-based saliency ROIs identified in our previous GLM, and asked whether mean activities in these ROIs significantly correlated with visual saliency. To this end we fit the new GLM with the visual saliency predictor to the voxel-wise average activity of these ROIs (rACC, striatum, anterior insula, and precentral gyrus), and conducted one-sample two-tailed *t* tests against zero of regression coefficients of the visual saliency predictor. Visual saliency coefficients were not significantly different from zero in any of the regions (mean ± SEM: rACC -0.016 ± 0.038, $p = 0.69$; striatum 0.011 ± 0.031 $p = 0.72$; anterior insula 0.036 ± 0.030, $p = 0.25$; precentral gyrus 0.020 ± 0.022, $p = 0.37$), again suggesting that these regions did not represent visual saliency.

Collectively, these findings show that neural activities in rACC, striatum, and anterior insula could not be attributed to visual saliency. Instead, their activations in our task were more consistent with the neural encoding of value-based saliency.

Supplementary Note 4: Univariate representation of category identity

In parallel with the investigation of univariate value and saliency signals, we also examined the univariate representation of category identity. Since the four categories in this experiment may differ in their overall value and saliency, it is important to control for the effect of these two covariates. We therefore constructed a general linear model with binary predictors for each of the four categories, while retaining the value and saliency regressors from the trial-by-trial ratings.

Using this model we conducted a whole-brain repeated-measures two-way ANOVA on the four category labels with modality (primary or secondary) and valence (positive or negative) as factors. Activation (*F* statistic) maps for the two main effects and their interaction were subject to statistical thresholding with the same criteria as all other analyses (per-voxel $p < 0.005$, cluster-size thresholding at FWE $p < 0.05$, threshold = 50 voxels), and the results are presented in Supplementary Table 2. Main effect of valence was observed in inferior frontal gyrus, lingual gyrus, parahippocampal gyrus, and middle frontal gyrus, while main effect of modality was located in inferior parietal lobule, precuneus, superior temporal gyrus, caudate, and parts of visual areas. Significant valence × modality interaction was observed in superior temporal gyrus, insula, middle temporal gyrus, cingulate gyrus, and lingual gyrus, suggesting that some of these areas encode information about specific categories.

We then searched directly for representations of specific category identities, using balanced contrasts of one category vs. the three other categories, namely category A > 1/3×(B + C + D). The same statistical threshold as above was applied (per-voxel $p <$ 0.005, cluster-size thresholding at FWE $p < 0.05$), and full results are presented in

Supplementary Table 3. Most of the category-selected activations were located in the visual cortex, likely due to differences in visual features of cue images of the categories. Outside of the visual areas, category-selective activations were relatively sparse, and included lateral prefrontal cortex (distinct areas for monetary gains or pleasant faces), superior temporal gyrus, dorsal anterior cingulate cortex, and insula (for electric shock).

To verify that these category-specific activations were driven by the stimuli of a single category, we further examined the mean beta coefficients for the binary category predictors in these regions (Supplementary Figure 5). Most non-visual areas revealed by the previous analysis indeed showed higher activity only for a single category, suggesting a category-specific coding. A more strict analysis, however, searching for whole-brain conjunction of all 3 pairwise contrasts for each category (category A > B & A > C & A > D), respectively, did not pass our statistical threshold (per-voxel $p < 0.005$, cluster-size thresholding at FWE $p < 0.05$, threshold = 82 voxels). Even with a more liberal threshold (per-voxel $p < 0.005$ uncorrected), no non-visual areas evolved from the conjunction analyses, except for the selective activation for the electric shock category in insula, dorsal anterior cingulate cortex, and inferior parietal cortex.

Supplementary Note 5: Region-of-interest representational similarity analysis (ROI RSA)

Results for this analysis are presented in Supplementary Figure 6.

Supplementary Note 6: Principal component analysis (PCA) of other value and saliency ROIs

Additional PCAs were performed on other value or saliency ROIs identified in the whole-brain univariate GLM analysis (Figures 2-4). The procedure was the same as described in Experimental Procedures. For all these regions, inspection of histograms of loading coefficients all revealed that the 1st PC resembled a mean-activity signal, while starting from the 2nd PC there were more complicated, uneven loading patterns. Results of mixed-effects regressions of the PCs on value, saliency, and the category dummy predictors are listed below.

Right OFC: 1st PC had significant value effect (p = 0.012), and no saliency (p = 0.35) or category (p > 0.22) effects; 2nd and 3rd PC had no significant effects.

Left OFC: No significant effects found for the PCs 1 to 3.

PPC: 1st PC had significant value effect (p = 0.0014), marginally significant saliency effect (p = 0.086) and no category effect (p > 0.21); 2nd and 3rd PC had no significant effects.

rACC: 1st PC had significant saliency effect (p = 0.0014) and no value (p = 0.31) or category effects (p > 0.79); 2nd and 3rd PC had no significant effects.

Striatum: 1st PC had significant saliency (p = 0.029) and value (p = 0.045) effect but no category effects (p > 0.28); 2nd and 3rd PC had no significant effects.

Anterior insula: 1st PC had significant saliency effect (p = 0.0057) and no value (p = 0.49) or category effects (p > 0.35); 2nd and 3rd PC had no significant effects.

Supplementary Discussion

Practical operationalization of value and saliency

Any study on valuation and its neural substrate has to deal with the operationalization of value and define how value will be measured (1). In this study we defined value as the self-reported unidimensional numerical estimate of the subjective pleasure elicited by cues predicting particular known outcomes, a concept equivalent to "utility" in economics. This, of course, has to rely on the assumption that participants have reasonably accurate insight of their own affective evaluations and that they report it faithfully, as far as the precision of the rating scale allows. One important alternative approach to value is revealed preferences through choice behavior. By choosing one option over another the individual reveals the preferential motivation elicited by a particular item or stimulus, which carries distinct properties compared with value as subjective pleasure (2, 3). While such an implicit approach may have certain advantages in revealing the real value on which choices are based, it relies heavily on the use of specific choice models, the theoretical assumptions of which are sometimes hard to verify. Instead, eliciting value with pleasantness rating removes the effect of active choice on the value signals, thus offering the benefit of simplicity in analysis and interpretation. It also minimizes the possibility that participants convert the value of options from different categories into units of a certain category (especially money). Furthermore, the various operationalizations of value should predict similar ranking orders of stimuli.

While value indicates how appetitive or aversive a stimulus is, saliency indicates the degree to which a particular stimulus captures attention or is important to the individual's current motivational goals. This loose definition may encompass both bottom-up

perceptual saliency (4-6) and top-down value-based saliency (7, 8). The latter can further include a number of cognitive processes, for example value-based attentional capture (9), emotional arousal (7, 10), motivational control (11), and goal-directed modulation of sensory processing (12). Indeed, the saliency-related brain areas we identified in the whole-brain univariate analysis included visual regions (mostly in the occipital cortex) as well as high-order cortical and sub-cortical areas, which have been previously implicated in saliency processes(13-16), and may receive input carrying saliency information from midbrain dopaminergic neurons (17-19). By incorporating visual saliency estimates of the cue images in an additional analysis, we have shown that saliency encoding in the visual areas was better explained by bottom-up visual saliency, while activities in rACC, striatum, and anterior insula were more consistent with top-down value-based saliency.

Practically, we computed value-based saliency as the squared difference between pleasantness rating and the pre-defined neutral point 5. The choice of this neutral point was consistent with the standard interpretation of the ratings that our participants were provided with, which also appeared on the screen next to the scale. Examination of the distribution of the mean pleasantness ratings in each subject (collapsing all categories and outcome magnitudes) showed that the subject-level mean ratings were indeed distributed within a relatively narrow range around 5, and the average of the subject-level mean ratings was 4.93. Therefore, without evidence of a systematic bias of the neutral point, it is conservative to adhere to the original prescription and calculate saliency estimates accordingly.

Supplementary Methods

Univariate representation of category identity

In addition to the main GLM (presented in Methods of the main text), to probe univariate representations of category identity, a second GLM was used. This GLM was similar to the first one, except it had four binary regressors for the cue period for non-actualized trials, one regressor for each category. All other regressors in the GLM remained the same, including all regressors of no interest. Representation of category identity was assessed by random-effects group analysis with balanced contrasts of one cue category vs. all three other categories, and the same per-voxel threshold and cluster-size thresholding were used as with the first GLM. The presence of trial-by-trial cue value and saliency regressors in this GLM ensured that any difference in activation between categories evolving from this analysis could not be attributed to differences in value or saliency between categories.

To control for the effect of valence (positive vs. negative) and modality (primary vs. secondary outcomes) in the encoding of category identity, we performed a whole-brain repeated-measures two-way ANOVA on the regression coefficients of the four binary category regressors in the GLM above. Valence and modality were included as two factors, and the global mean of the beta maps was incorporated as a covariate. The resulting F maps for the two main effects and their interaction were subject to statistical thresholding (per-voxel $p < 0.005$, cluster-size thresholding at FWE $p < 0.05$ with simulation of 1000 random samples).

Excluding the potential confound of hand movement for the univariate saliency signal

A control analysis was performed for the saliency-related activations and summarized below (also see Supplementary Results, and Supplementary Figure 3). Since participants needed to move the cursor from the central starting position on the scale to the desired number, the number of button presses increased as the rating deviated more from the neutral rating 5. As a result, saliency estimates based on participants' trial-by-trial ratings were correlated with hand movement and/or the time it took to register the response. To address these potential confounds, we performed an additional control analysis utilizing the second rating (for the outcomes) in the task. A signal simply driven by hand movement should show consistent correlation with key presses in both the cue and the outcome periods. In this spirit, we directly modeled the number of key presses required for each rating in both periods of every trial in a GLM, with which we searched for signals that met this criterion and contrasted them with saliency-relate activities.

For assessing the potential effect of hand movement and/or reaction time in saliency regions, we created a new GLM, with the following predictors:

1) A binary indicator for the cue period (including both actualized and non-actualized cues);

2) A parametric modulator for the cue period, indicating the number of key presses required for the response (based on ratings, assuming participants go directly from the start position 5 to the desired rating);

3) A binary indicator for the outcome period (including both delivered and undelivered outcomes);

4) A parametric modulator for the outcome period, indicating the number of key presses required for the response (based on ratings, assuming participants go directly from the start position 5 to the desired rating);

As usual, a constant and the 6 motion parameters were added. All predictors were convolved with a standard canonical hemodynamic response function.

Compared with the main GLM for identifying value and saliency signals, this GLM has the following features. First, this GLM explicitly modeled the number of key presses for the participants to register the response. This both enabled a more fine-grained modeling of hand movement/RT and eliminated differences between saliency and the number of key presses. Second, including all trials (reinforced and non-reinforced) in this GLM ensured that there were equal numbers of trials in both periods, and therefore the statistical power should be the same. Third, although the assumption that participants went straight from the starting position 5 to the desired rating without going back and forth might not hold true for all trials, making this assumption actually worked against our position on this issue – should there have been a substantial proportion of trials in which this assumption was false, there would be much less correlation between hand movement/RT and saliency.

This GLM was then fit to the mean BOLD time courses of the saliency ROIs we identified, including rACC, striatum, precentral gyrus, and anterior insula. Paired *t* tests were conducted to compare the mean regression coefficients for the number of key presses in the cue period and in the outcome period.

Separating the neural substrates of value-based saliency and visual saliency

To further examine the neural substrates of perceptual (visual) saliency and of value-based saliency, we fit a GLM incorporating visual saliency of the cue stimuli as a separate regressor. To quantify the visual saliency of the cue stimuli in the paradigm, we applied a visual saliency model proposed by a recent study on the computational modeling of human visual perception (66) to the 16 cue stimuli. This is a biologically inspired model, motivated by the sparse coding observed in primary visual cortex and by feature-based attentional signals in V4 and MT, taking into account multiple visual features, e.g. luminosity, color, edges, etc. Most notably, this model achieves superior accuracy in predicting fixation locations and eye movements in image and video viewing of human participants, and therefore could be used as a reliable way of predicting visual saliency of images. For any image fed into this model, a saliency map is generated, with pixel-by-pixel visual saliency values predicted by the model. In order to ensure the estimation of visual saliency for all 16 stimuli on a common scale, we generated 1000 random 4-by-4 collage images of the 16 stimuli, shuffling the locations of specific stimuli. The mean predicted visual saliency of the square area covering each stimulus, averaged across all pixels in the area and then across 1000 collage images, was then used as an estimate for the visual saliency of this particular stimulus.

With these visual saliency estimates, we then constructed a new GLM for controlling the potential confound of visual saliency. This GLM was almost identical to the main GLM used to search for value and (value-based) saliency signals in the whole brain, with the visual saliency estimates as one additional parametric modulator for the cue period (after convolution with a standard canonical hemodynamic response function). Therefore, for non-actualized trials, the cue was modeled by a binary regressor and *three* parametric

regressors modulated by trial-by-trial (1) value, (2) value-based saliency, and (3) visual saliency estimates. Given the potential similarity between value-based saliency and visual saliency, we examined the correlation between predictors of value-based saliency and visual saliency. Across all participants, the mean R^2 was 0.243 ± 0.050 (SEM), which was in a reasonable range so that the design matrices of the GLM were not ill-defined. Similarly, the mean variance inflation factors (VIF) for the value-based saliency and visual saliency predictors were 1.50 ± 0.12 (SEM) and 1.78 ± 0.71, respectively, indicating relatively low multi-collinearity of the design matrices.

Both whole-brain analysis and ROI analysis were performed with this GLM. In the whole-brain analysis, activations corresponding to value-based saliency and visual saliency were identified with the same statistical threshold we used throughout the study (per-voxel $p < 0.005$, cluster-size thresholding at FWE $p < 0.05$ with simulation of 1000 samples). To further examine the specificity of value-based and visual saliency representation, we also ran a direct contrast of value-based saliency vs. visual saliency, and applied the statistical threshold above. Results of these analyses are presented in Supplementary Data, Supplementary Figure 4, and Supplementary Table 1.

For the ROI-based analysis, the above GLM was fit to the mean activation in the four saliency regions presented in the main text (rACC, striatum, precentral gyrus, and anterior insula). One-sample *t* tests of the regression coefficients for visual saliency were performed for these four ROIs, separately, as a further confirmation that these ROIs represented value-based saliency, rather than visual saliency.

Region-of-interest representational similarity analysis (ROI RSA)

RSA on ROIs defined by the univariate GLM analysis was largely similar to whole-brain searchlight RSA. The dimensions of multi-voxel patterns in ROI RSA were determined by the number of voxels in the ROI under consideration, instead of being fixed as in the searchlight analysis. Neural RDMs were again computed using the Pearson correlational distance described in the main text. They were then compared to the candidate model RDMs, including the category, value, saliency, and category-mean-value models. The same statistical procedure (detailed in Experimental Procedures) was followed to determine the significance (*p* value) for each candidate model.

Statistical inference was also performed to test whether two candidate RDMs differ in their contribution to the neural RDM of the ROI under consideration. The difference between the RDM correlations in each participant was computed, and a two-sided signed-rank test across participants was performed. Multiple testing was corrected for by controlling the false-discovery rate (20, 21).

Supplementary References

1. O'Doherty JP (2014) The problem with value. *Neurosci Biobehav R* 43:259-268.
2. Berridge KC (1996) Food reward: Brain substrates of wanting and liking. *Neurosci Biobehav R* 20(1):1-25.
3. Rangel A, Camerer C, & Montague PR (2008) A framework for studying the neurobiology of value-based decision making. *Nat Rev Neurosci* 9(7):545-556.
4. Wang LH, Yu HB, & Zhou XL (2013) Interaction between value and perceptual salience in value-driven attentional capture. *J Vision* 13(3).
5. Lee TS, Yang CF, Romero RD, & Mumford D (2002) Neural activity in early visual cortex reflects behavioral experience and higher-order perceptual saliency. *Nat Neurosci* 5(6):589-597.
6. Zink CF, Pagnoni G, Chappelow J, Martin-Skurski M, & Berns GS (2006) Human striatal activation reflects degree of stimulus saliency. *Neuroimage* 29(3):977-983.
7. Litt A, Plassmann H, Shiv B, & Rangel A (2011) Dissociating Valuation and Saliency Signals during Decision-Making. *Cereb Cortex* 21(1):95-102.
8. Kahnt T, Park SQ, Haynes JD, & Tobler PN (2014) Disentangling neural representations of value and salience in the human brain. *P Natl Acad Sci USA* 111(13):5000-5005.
9. Anderson BA, Laurent PA, & Yantis S (2011) Value-driven attentional capture. *P Natl Acad Sci USA* 108(25):10367-10371.
10. Maunsell JHR (2004) Neuronal representations of cognitive state: reward or attention? *Trends Cogn Sci* 8(6):261-265.
11. Locke HS & Braver TS (2008) Motivational influences on cognitive control: Behavior, brain activation, and individual differences. *Cogn Affect Behav Ne* 8(1):99-112.
12. Serences JT (2008) Value-Based Modulations in Human Visual Cortex. *Neuron* 60(6):1169-1181.
13. Petersen SE & Posner MI (2012) The Attention System of the Human Brain: 20 Years After. *Annual Review of Neuroscience, Vol 35* 35:73-89.
14. Rudebeck PH*, et al.* (2014) A role for primate subgenual cingulate cortex in sustaining autonomic arousal. *P Natl Acad Sci USA* 111(14):5391-5396.
15. Zink CF, Pagnoni G, Martin ME, Dhamala M, & Berns GS (2003) Human striatal response to salient nonrewarding stimuli. *J Neurosci* 23(22):8092-8097.
16. Zink CF, Pagnoni G, Martin-Skurski ME, Chappelow JC, & Berns GS (2004) Human striatal responses to monetary reward depend on saliency. *Neuron* 42(3):509-517.
17. Steinfels GF, Heym J, Strecker RE, & Jacobs BL (1983) Behavioral-Correlates of Dopaminergic Unit-Activity in Freely Moving Cats. *Brain Res* 258(2):217-228.
18. Steinfels GF, Heym J, Strecker RE, & Jacobs BL (1983) Response of Dopaminergic-Neurons in Cat to Auditory-Stimuli Presented across the Sleep Waking Cycle. *Brain Res* 277(1):150-154.
19. Bromberg-Martin ES, Matsumoto M, & Hikosaka O (2010) Dopamine in motivational control: rewarding, aversive, and alerting. *Neuron* 68(5):815-834.
20. Benjamini Y & Hochberg Y (1995) Controlling the False Discovery Rate - a Practical and Powerful Approach to Multiple Testing. *J Roy Stat Soc B Met* 57(1):289-300.
21. Benjamini Y, Drai D, Elmer G, Kafkafi N, & Golani I (2001) Controlling the false discovery rate in behavior genetics research. *Behav Brain Res* 125(1-2):279-284.
